# Food Problem Notes

**Published:** 1918-03-09

**Authors:** 


### Food Problem Notes.

Saving of waste—not by reduction of rations—in the Army has effected a saving of £800,000, of which £225,000 has been returned in improved food and wages to cooks, and the remainder to the country. It has also produced enough tallow to supply the Service with soap and release some also for public sale, and a quantity of crude glycerine which has been sold to the Ministry of Munitions at £60 per ton, as against £300 for imported glycerine.

---

An adult may very well save 5 oz. sugar from the weekly ration for jam. Glucose for this purpose need not be considered unwholesome; it is prepared from starch by the action of sulphuric acid.

---

To save butter and margarine use 1 lb. and 1 pint of milk with one tablespoon of gelatine powder. Melt, mix, beat till the blend begins to "firm," then make pats.

---

Let us interest ourselves in the *romance* of food. We speak of War bread, Americans of Victory bread. The potato was popularised in France, and thence in England, by the enthusiasm of one Eugène Parmentier, fed on potatoes while a prisoner in Germany. The French king gave *cachet* by wearing the flower in his buttonhole.

---

The present writer would add, let us encourage in children an enthusiasm for mother-care. Rations for the whole family will make small dishes unless substitutes are vigorously used. "What boy in this school saw that his mother had a proper helping at dinner to-day?"

---

Soldiers on last leave will get the equivalent of Home service meat rations, about 8 oz. per day. On lengthy leave they will be treated as civilians. On journeys of more than six hours' length they will get coupons available at huts, railway buffets, etc.

---

In connection with the Note on "Rations in Sanatoria" (see p. 478), the following is the weekly allowance of each article "as purchased":—

| — | For Persons without much Constitutional Disturbance. | | For Persons with Constitutional Disturbance. | |
|---|---|---|---|---|
| | Men | Women | Men | Women |
| Milk ... ... ... | 14 pints | 14 pints | 21 pints | 21 pints |
| Meat (including suet) ... | 3½ lb. | 3 lb. | 3½ lb. | 3 lb. |
| Bacon ... ... ... | ½ lb. | ½ lb. | ½ lb. | ½ lb. |
| Fish ... ... ... | ½ lb. | ½ lb. | 1 lb. | 1 lb. |
| Cheese ... ... ... | ½ lb. | ½ lb. | 4 oz. | 4 oz. |
| Oatmeal ... ... ... | ½ lb. | ½ lb. | ½ lb. | ½ lb. |
| Pulses ... ... ... | ½ lb. | ½ lb. | — | — |
| Bread ... ... ... | 4 lb. | 3 lb. | 3 lb. | 3 lb. |
| Flour ... ... ... | ½ lb. | ½ lb. | ½ lb. | ½ lb. |
| Potatoes ... ... ... | 5 lb. | 4 lb. | 2 lb. | 2 lb. |
| Cereals ... ... ... | ¾ lb. | ¾ lb. | ¾ lb. | ¾ lb. |
| Sugar ... ... ... | ½ lb. | ½ lb. | ½ lb. | ½ lb. |
| Jam, syrup, &c- ... | ¾ lb. | ¾ lb. | ¾ lb. | ¾ lb. |
| Margarine and other fats | 10 oz. | 10 oz. | 10 oz. | 10 oz. |

At any time it may be necessary to substitute equivalent amounts of other foodstuffs for not more than 1 lb. of meat or 7 pints of milk per week.

